# Echocardiographic parameters and renal outcomes in patients with preserved renal function, and mild- moderate CKD

**DOI:** 10.1186/s12882-018-0975-5

**Published:** 2018-07-11

**Authors:** Thomas A. Mavrakanas, Aisha Khattak, Karandeep Singh, David M. Charytan

**Affiliations:** 1 Renal Division, Brigham & Women’s Hospital, Harvard Medical School, Boston, MA USA; 2 Division of Nephrology, Department of Medicine, McGill University, Montreal, Canada; 3 Division of Renal Disease and Hypertension, Department of Internal Medicine, The University of Texas Health Science Center, Houston, TX USA; 4 Departments of Learning Health Sciences and Internal Medicine, University of Michigan Medical School, Ann Arbor, MI USA

**Keywords:** Echocardiography, Chronic kidney disease, Doubling of serum creatinine, Dialysis, Kidney transplantation, Mortality

## Abstract

**Background:**

Echocardiographic characteristics across the spectrum of chronic kidney disease (CKD) have not been well described. We assessed the echocardiographic characteristics of patients with preserved renal function and mild or moderate CKD referred for echocardiography and determined whether echocardiographic parameters of left ventricular (LV) and right ventricular (RV) structure and function were associated with changes in renal function and mortality.

**Methods:**

This retrospective cohort study enrolled all adult patients who had at least one trans-thoracic echocardiography between 2004 and 2014 in our institution. The composite outcome of doubling of serum creatinine or initiation of maintenance dialysis or kidney transplantation was the primary outcome. Mortality was the secondary outcome.

**Results:**

29,219 patients were included. Patients with worse renal function had higher prevalence of structural and functional LV and RV abnormalities. Higher estimated glomerular filtration rate (eGFR) was independently associated with preserved LV ejection fraction, preserved RV systolic function, and lower LV mass, left atrial diameter, pulmonary artery pressure, and right atrial pressure, as well as normal RV structure. 1041 composite renal events were observed. 8780 patients died during the follow-up. Pulmonary artery pressure and the RV, but not the LV, echocardiographic parameters were independently associated with the composite renal outcome. In contrast, RV systolic function, RV dilation or hypertrophy, LV ejection fraction group, LV diameter quartile, and pulmonary artery pressure quartile were independently associated with all-cause mortality.

**Conclusions:**

Echocardiographic abnormalities are frequent even in early CKD. Echocardiographic assessment particularly of the RV may provide useful information for the care of patients with CKD.

**Electronic supplementary material:**

The online version of this article (10.1186/s12882-018-0975-5) contains supplementary material, which is available to authorized users.

## Background

Chronic kidney disease (CKD) is highly prevalent among patients with congestive heart failure (CHF) and is associated with higher mortality rates [1]. In the CHARM trials, for example, an estimated glomerular filtration rate (eGFR) < 60 ml/min/1.73m^2^ was an independent predictor of cardiovascular death or admission for decompensated heart failure [2].

Echocardiography can identify abnormalities in heart function or structure that are associated with kidney dysfunction [3]. Abnormal left ventricular (LV) geometry, lower mid-wall fractional shortening, and higher LV mass index have been described among patients with CKD compared with patients with preserved renal function in a cohort of participants with heart failure with preserved ejection fraction [3]. In other studies, echocardiographic parameters predicted cardiovascular events among CKD patients, although there was no net reclassification improvement when these parameters were added to a model including clinical and laboratory variables [4].

Although these data are intriguing, the echocardiographic characteristics of patients across the spectrum of cardiac function – with both preserved or reduced LV ejection fraction and with or without CHF – have not been adequately described across different eGFR stages, especially in patients with mildly decreased eGFR. It is also uncertain whether echocardiographic parameters predict CKD progression. The objective of this study was to describe the echocardiographic characteristics of patients with preserved renal function and mild or moderate CKD referred for echocardiography, and to assess associations of echocardiographic parameters with changes in renal function.

## Methods

### Study population

Patients were included if they had at least one trans-thoracic echocardiography with tissue Doppler imaging at a Partners affiliated healthcare facility between January 1st 2004 and January 6th 2014, were ≥ 18 years old at the time of echocardiography, and had at least one eGFR between 30 and 120 ml/min/1.73m^2^ during this time period. For patients who had more than one echocardiogram, the first one was used.

Patients on maintenance dialysis or a history of kidney or heart transplant were excluded, as were those without creatinine values from 365 days before to 90 days after echocardiography.

The study was designed to capture patients with preserved renal function and mild or moderate CKD (eGFR≥90, 60–89, 30–59 ml/min/1.73m^2^).

### Data collection

The Research Patient Data registry (RPDR), a registry of all patients followed at a Partners healthcare facility, was used to retrieve all encounters, medication prescriptions, laboratory tests, and echocardiography results conducted during this time period.

To avoid misdiagnosis of acute kidney injury at the time of baseline echocardiography as CKD, the average baseline creatinine was calculated using all the available creatinines for each patient from 365 days prior to 90 days after the index echocardiogram (16 values on average, median of 7). Baseline eGFR was calculated using the CKD-EPI equation.

### Comorbidities and baseline data

Information on comorbidities was extracted using condition-specific diagnostic codes (International Classification of Diseases, 9th revision-Additional file 1 Table S1). Comorbidities were diagnosed on the basis of relevant inpatient or outpatient diagnoses preceding the echocardiography date. As many echocardiograms are performed during a first admission for decompensated heart failure, any patient with a first CHF diagnostic code in the 30 days after the echocardiogram was considered to have baseline CHF.

The following baseline laboratory data were extracted (the values closest to the echocardiogram date, from 365 days before to 90 days after echocardiography): hemoglobin, albumin, calcium, phosphorus, urine albumin to creatinine ratios, and brain natriuretic peptide (BNP) levels.

We determined the use of angiotensin converting enzyme inhibitors (ACEI), angiotensin receptor blockers (ARB), β-blockers, statins, aspirin, clopidogrel, and warfarin by matching the most recent outpatient medication lists with generic and brand name drugs.

### Echocardiograms

The following parameters were extracted from the echocardiography reports using automated text processing: LV ejection fraction, LV diastolic diameter, LV posterior wall thickness, interventricular septal thickness in diastole, LV mass (area-length method), LV mass index (corrected for body surface area), pulmonary artery pressure, right atrial pressure, left atrium diameter, aortic root diameter, concentric hypertrophy and regional wall motion abnormalities, right ventricular (RV) systolic function (preserved or reduced), and RV hypertrophy or dilatation (presence or absence). All echocardiogram reports were acquired and recorded using an American Society of Echocardiography-recommended scanning protocol with standard techniques. Although we initially planned to examine associations of diastolic dysfunction with CKD progression, tissue Doppler imaging results were image-embedded and not readily extractable.

We randomly reviewed 100 patient records to check the accuracy of automated extraction of diagnoses, medication, and echocardiographic parameters. Specificity was 100% and sensitivity was between 95 and 97% for the different echocardiographic parameters (most available information was captured using regular expression searches).

### Outcomes

We retrospectively searched in the registry for available creatinine values or diagnostic codes post index echocardiography. The composite of doubling of serum creatinine, maintenance dialysis or kidney transplantation was the primary outcome because it is a standard, FDA-validated outcome. Mortality was the secondary outcome. We also analyzed associations between echocardiographic parameters and eGFR, using the latter as the exposure.

Clinical outcomes were identified from RPDR using diagnostic codes. The RPDR is continuously matched to the social security death index, a national database including all deaths in the United States. For the outcome of doubling of serum creatinine, creatinine values starting 30 days after the first doubling as well as the last available creatinine were screened for repeated/confirmed doubling to rule out potential AKI. Patients were censored at the last clinical visit or lab test.

### Statistical analysis

Baseline characteristics across eGFR groups were compared using the χ^2^ test for categorical variables, and one-way analysis of variance or non-parametric trend tests for continuous variables.

In order to study the association between echocardiographic parameters and eGFR, we used linear regression for continuous echocardiographic variables or logistic regression for binary variables. Normality, linearity, and homoscedasticity assumptions were confirmed using graphical methods. The following covariates were included in multivariate models on the basis of known associations with the outcomes of interest, clinical intuition, and availability in the dataset: age, sex, race, history of hypertension, diabetes, coronary disease, CHF, and use of ACEI or ARB.

In order to assess event-free survival, the cohort was divided into quartiles for LV diastolic diameter, LV mass, LV mass index, left atrium diameter, and pulmonary artery pressure. Categorical variables or variables reported in intervals rather than as true continuous measurements were assessed in binary categories (RV systolic function, RV hypertrophy and dilation, and right atrial pressure) except for LV ejection fraction where the following cutoffs were used: < 25%, 25–39%, 40–54%, ≥55%. The incidence of the composite renal outcome was calculated for each quartile. The χ^2^ test was used to compare the different groups. To assess associations of echocardiographic parameters with clinical outcomes, multivariable Cox proportional hazards regression was used. Proportional hazards assumptions were verified using graphical methods. Patients without available creatinine values or diagnostic codes for dialysis or transplantation were considered to have stable renal function. The following parameters were included in the baseline model: age, sex, race, baseline eGFR, history of hypertension, diabetes, coronary artery disease, CHF, and use of ACEI or ARB. The various echocardiographic parameters were then added to the model. Patients with missing echocardiographic parameters were excluded from the respective survival analysis.

We conducted three sensitivity analyses in the following subgroups: i) outpatients only—in order to exclude individuals with echocardiograms during an acute cardiac event; ii) individuals without CHF at baseline; iii) individuals with available follow-up creatinine values or diagnostic codes.

*P*-values < 0.05 were considered statistically significant. SPSS software (version 20.0, SPSS Inc., Chicago, IL, USA) was used for all statistical analyses.

## Results

### Echocardiographic parameters in different eGFR groups

We identified 42,446 patients with at least one echocardiogram and one eGFR value between 30 and 120 ml/min/1.73m^2^. Of these, 31,173 had at least one creatinine value available from 365 days before to 90 days after the echocardiogram. 492 patients with a transplanted heart or kidney, 268 patients on dialysis at baseline, and 1194 patients who had a baseline eGFR < 30 ml/min/1.73 m^2^ were excluded from the main analysis. The characteristics of the 29,219 patients with an eGFR ≥30 ml/min/1.73m^2^ at baseline are depicted in Table 1. Mean time between echocardiography and serum creatinine was 2.3 ± 0.1 days. Patients with worse renal function were older, more commonly white, had a higher prevalence of hypertension, diabetes, coronary artery disease, or heart failure, higher urine albumin to creatinine ratios, and higher potassium and phosphorus levels. BNP levels were also higher with increasing creatinine.

**Table 1:** Baseline characteristics of the study patients

Characteristic	eGFR 90–120		eGFR 60–89		eGFR 30–59	
	N	Result	N	Result	N	Result
Patients		9864 (34%)		12,731 (43%)		6624 (23%)
Outpatients		4558 (46%)		6124 (48%)		2448 (37%)
Age (years)	9864	50 ± 14	12,731	64 ± 13	6624	72 ± 12
Male sex	9864	4797 (49%)	12,731	6608 (52%)	6624	3281 (50%)
African American	9864	1168 (12%)	12,731	949 (8%)	6624	523 (8%)
Hypertension	9864	3480 (35%)	12,731	6884 (54%)	6624	4091 (62%)
Diabetes mellitus	9864	1308 (13%)	12,731	2307 (18%)	6624	1902 (29%)
CAD	9864	3087 (31%)	12,731	5984 (47%)	6624	3762 (57%)
CHF	9864	1733 (18%)	12,731	3369 (27%)	6624	3081 (47%)
COPD	9864	286 (3%)	12,731	454 (4%)	6624	319 (5%)
PE	9864	520 (5%)	12,731	452 (4%)	6624	257 (4%)
ACEI	8953	1614 (18%)	11,642	3278 (28%)	6175	2222 (36%)
ARB	8953	330 (4%)	11,642	928 (8%)	6175	810 (13%)
β-blocker	8953	3618 (40%)	11,642	6190 (53%)	6175	3902 (63%)
Statin	8953	2298 (26%)	11,642	5077 (44%)	6175	3317 (54%)
Aspirin	8953	2993 (33%)	11,642	5287 (45%)	6175	3373 (55%)
Clopidogrel	8953	659 (7%)	11,642	1325 (11%)	6175	844 (14%)
Warfarin	8953	940 (11%)	11,642	1852 (16%)	6175	1168 (19%)
Creatinine (μmol/l)	9864	65 (57–74)	12,731	82 (73–94)	6624	115 (100–134)
eGFR (ml/min/1.73m^2^)	9864	104 ± 12	12,731	76 ± 9	6624	47 ± 8
Albuminuria stage:						
- A1	348	213 (61%)	544	333 (61%)	472	188 (40%)
- A2	348	100 (29%)	544	159 (29%)	472	191 (41%)
- A3	348	35 (10%)	544	52 (10%)	472	93 (20%)
Hb (g/l)	7115	123 ± 23	8458	127 ± 21	4301	118 ± 21
Albumin (g/l)	7331	38 ± 7	9261	39 ± 6	5183	37 ± 6
K^+^ (mmol/l)	9522	4.0 ± 0.4	12,312	4.0 ± 0.4	6448	4.1 ± 0.5
Calcium (mmol/l)	8491	2.20 ± 0.18	10,961	2.23 ± 0.15	5796	2.23 ± 0.18
Phos (mmol/l)	3974	1.0 (0.8–1.2)	4188	1.0 (0.9–1.2)	2917	1.1 (0.9–1.2)
BNP (ng/L)	906	90 (27–265)	1610	188 (62–480)	1519	349 (137–745)

Results are presented as number (percentage), mean ± standard deviation, or median (interquartile range). eGFR, estimated glomerular filtration rate; N, number of patients with available data; CAD, coronary artery disease; CHF, congestive heart failure; COPD, chronic obstructive lung disease; PE, pulmonary embolism (acute or chronic); ACEI, angiotensin converting enzyme inhibitor; ARB, angiotensin receptor blocker; A1: albuminuria < 30 mg/g of creatinine; A2: albuminuria 30–300 mg/g of creatinine; A3: albuminuria > 300 mg/g of creatinine; Hb, hemoglobin; K^+^, potassium; Phos, phosphorus; BNP, brain natriuretic peptide. Percentages are within eGFR group and exclude missing values. The three eGFR groups were statistically different (P value for trend < 0.001 for all parameters except sex)

Patients with worse renal function had lower LV ejection fraction, higher prevalence of RV systolic impairment, higher LV mass index, higher LV and left atrium diameter, more regional wall motion abnormalities, and higher prevalence of RV hypertrophy or dilated RV **(**Table 2**)**. They also had higher pulmonary artery pressures, and higher prevalence of elevated right atrial pressure. eGFR was independently associated with all echocardiographic parameters analyzed in multi-variable models (Additional file 2 Table S2**)**.

**Table 2:** Echocardiographic characteristics of the study patients

	eGFR 90–120		eGFR 60–89		eGFR 30–59	
Characteristic	N	Result	N	Result	N	Result
Preserved EF	9161	7604 (83%)	11,730	8975 (77%)	6192	4058 (66%)
LVEF (%)	9161	60 (55–60)	11,730	60 (55–60)	6192	55 (45–60)
LVd (cm)	7576	4.66 ± 0.72	9562	4.64 ± 0.80	5411	4.70 ± 0.93
LVPW (cm)	5247	0.98 ± 0.22	6141	1.04 ± 0.23	3431	1.09 ± 0.25
IVSd (cm)	6918	1.02 ± 0.24	8759	1.10 ± 0.25	4975	1.15 ± 0.26
LVM (g)	3853	161 (126–205)	4229	174 (135–223)	2377	186 (146–243)
LVMi (g/m^2^)	2857	85 (69–103)	3204	92 (74–115)	1705	102 (81–128)
LAd (cm)	7299	3.66 ± 0.72	9277	3.92 ± 0.78	5245	4.14 ± 0.83
PAP (mmHg)	5309	22 (18–28)	7385	25 (19–31)	4359	28 (22–37)
RAP > 6 cm H_2_O	3831	283 (7%)	4400	449 (10%)	2317	372 (16%)
Impaired RV systolic function	7810	430 (6%)	9887	789 (8%)	5376	717 (13%)
Increased RV diameter	7982	728 (9%)	10,148	1114 (11%)	5555	907 (16%)
RV hypertrophy	4990	117 (2%)	5888	171 (3%)	2840	172 (6%)
ARd (cm)	6946	3.09 ± 0.47	8927	3.18 ± 0.47	5052	3.18 ± 0.48
RWMA	6320	348 (6%)	7366	718 (10%)	3377	540 (16%)

Results are presented as number (percentage), mean ± standard deviation, or median (interquartile range). N, number of patients with available data; LVEF, left ventricular ejection fraction; EF, ejection fraction; LVd, left ventricular diastolic diameter; LVPW, left ventricular posterior wall thickness; IVSd, interventricular septal thickness in diastole; LVM, left ventricular mass (area-length method); LVMi, left ventricular mass index (corrected for body surface area); LAd, left atrium diameter; PAP, pulmonary artery pressure; RAP, right atrial pressure; RV, right ventricle; ARd, aortic root diameter; RWMA, regional wall motion abnormalities. Percentages exclude missing values. The three eGFR groups were statistically different (P value for trend < 0.001 for all parameters except LVd; *p* = 0.01 for LVd)

### Echocardiographic parameters in patients with and without history of CHF

A total of 8183 patients had history of CHF: 1733 (21%) with an eGFR ≥90, 3369 (41%) with an eGFR of 60–89, and 3081 (38%) with an eGFR < 60 ml/min/1.73m^2^. The baseline and echocardiographic characteristics according to eGFR groups of patients with CHF are shown in Additional file 3: Table S3 and Additional file 4: Table S4.

In the subgroup of patients without CHF, prevalence of various comorbidities at baseline was lower compared with patients with CHF. In those without CHF, right atrial pressure levels were similar in all three eGFR groups. For all the other echocardiographic parameters, a significant linear trend was identified across the different eGFR groups but the prevalence of RV or LV systolic dysfunction was lower (Additional file 5: Table S5 and Additional file 6: Table S6).

### Renal outcomes

At least one follow-up creatinine (beyond day 90 from the index echocardiogram) or diagnostic code for dialysis/transplantation were available for 20,008 patients (68%). Median follow up was 48 months (interquartile range 20–87 months). A total of 1041 composite renal events were observed: 691 events with the creatinine doubling, 273 initiations of dialysis, and 77 kidney transplantations; 282 occurred in patients with an eGFR ≥90 (0.86 events per 100 patients-years), 348 in patients with eGFR between 60 and 89 (0.73 events per 100 patients-years), and 411 in patients with an eGFR < 60 ml/min/1.73m^2^ (1.79 events per 100 patients-years).

**Fig. 1:**
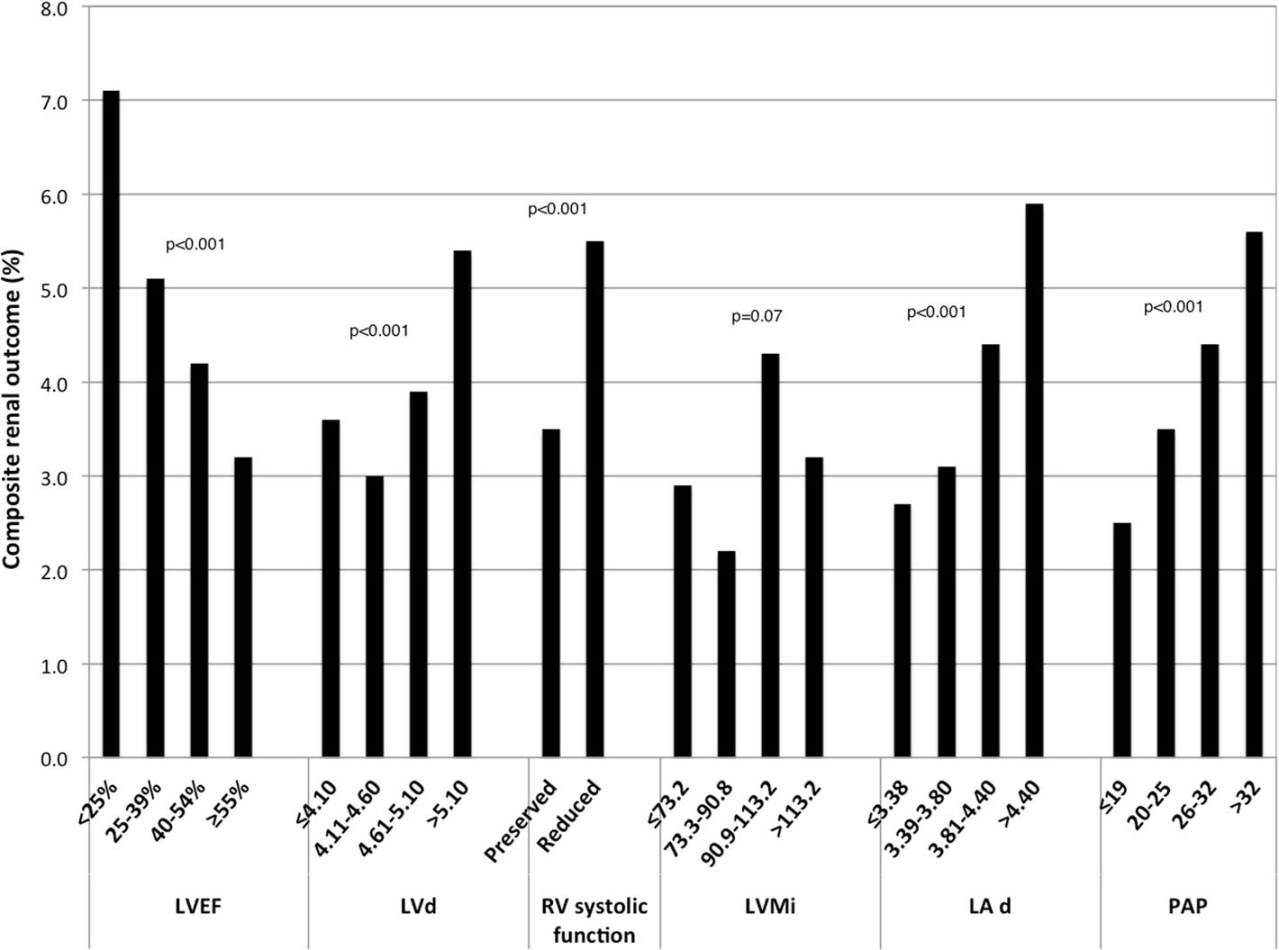
Percentage of patients with CKD progression by category of echocardiographic parameters. The incidence of the composite renal outcome (doubling of serum creatinine or initiation of maintenance dialysis or kidney transplantation) was calculated for each quartile of LVd, LVM, LVMi, LAd, and PAP. For LVEF, the following cutoffs were used: < 25%, 25–39%, 40–54%, ≥55%. *P* values are for trend. LVEF, left ventricular ejection fraction; LVd, left ventricular diastolic diameter; RV, right ventricular; LVMi, left ventricular mass index (corrected for body surface area); LA d, left atrium diameter; PAP, pulmonary artery pressure. P for trend provided

The number of composite renal events that occurred in the 1st, 2nd, 3rd, and 4th quartile of each echocardiographic parameter is depicted in Fig. 1. An increase was observed in higher quartiles of LV and left atrial diameter, LV mass and pulmonary artery pressure but not of LV mass index. Lower LV ejection fraction groups also had higher renal event rates. However, in adjusted models, most LV parameters were not independently associated with the composite renal outcome, with the exception of the second quartile of LV diastolic diameter (Table 3). In contrast, pulmonary artery pressure and RV systolic function and dilation were independent predictors of the composite renal outcome. The adjusted HR for impaired RV systolic function was 1.51 (95% CI 1.21–1.88, *p* < 0.001), and for RV dilation 1.59 (95% CI 1.32–1.92, p < 0.001).

**Table 3:** Adjusted associations of echocardiographic parameters with composite renal outcomes and mortality

	Parameter	Number of renal events	Adjusted HR (95% CI) - Renal outcomes	*p*	Number of deaths	Adjusted HR (95% CI) - Mortality	*p*
LVEF	Group 1 vs. 4	67 vs. 649	1.18 (0.88–1.59)	0.26	423 vs. 5602	1.46 (1.31–1.63)	< 0.001
	Group 2 vs. 4	91 vs. 649	1.06 (0.82–1.36)	0.66	803 vs. 5602	1.27 (1.17–1.38)	< 0.001
	Group 3 vs. 4	160 vs. 649	1.05 (0.87–1.26)	0.64	1403 vs. 5602	1.09 (1.02–1.16)	0.007
LVd	Quartile 2 vs. 1	178 vs. 208	0.77 (0.63–0.95)	0.02	1805 vs. 2211	0.83 (0.78–0.89)	< 0.001
	Quartile 3 vs. 1	211 vs. 208	0.97 (0.79–1.19)	0.78	1553 vs. 2211	0.80 (0.75–0.86)	< 0.001
	Quartile 4 vs. 1	292 vs. 208	1.02 (0.83–1.26)	0.83	1872 vs. 2211	0.84 (0.78–0.90)	< 0.001
LVMi	Quartile 2 vs. 1	42 vs. 56	0.72 (0.47–1.08)	0.11	450 vs. 458	0.93 (0.81–1.06)	0.29
	Quartile 3 vs. 1	84 vs. 56	1.29 (0.90–1.84)	0.17	551 vs. 458	0.97 (0.85–1.11)	0.65
	Quartile 4 vs. 1	63 vs. 56	0.81 (0.54–1.22)	0.31	626 vs. 458	0.95 (0.83–1.08)	0.41
PAP	Quartile 2 vs. 1	167 vs. 115	1.31 (1.02–1.67)	0.03	1452 vs. 1090	1.11 (1.02–1.20)	0.02
	Quartile 3 vs. 1	157 vs. 115	1.67 (1.30–2.15)	< 0.001	1374 vs. 1090	1.26 (1.16–1.37)	< 0.001
	Quartile 4 vs. 1	223 vs. 115	2.26 (1.77–2.88)	< 0.001	2062 vs. 1090	1.73 (1.60–1.88)	< 0.001
RV systolic function	Reduced vs. preserved	106 vs. 743	1.51 (1.21–1.88)	< 0.001	881 vs. 6494	1.61 (1.49–1.73)	< 0.001
RV hypertrophy	Present vs. absent	26 vs. 417	1.33 (0.88–2.02)	0.18	204 vs. 3971	1.17 (1.01–1.35)	0.04
RV dilation	Present vs. absent	149 vs. 740	1.59 (1.32–1.92)	< 0.001	1189 vs. 6414	1.45 (1.36–1.55)	< 0.001

Adjusted Cox models. Hazard ratios are adjusted for age, sex, race, baseline eGFR, history of hypertension, diabetes, CAD, or CHF, and use of ACEI and/or ARB. Composite renal outcome includes doubling of serum creatinine or initiation of maintenance dialysis or kidney transplantation. For LVEF, the following cutoffs were used: < 25%, 25–39%, 40–54%, ≥55%. HR, hazards ratio; CI, confidence interval; AA, African American race; eGFR, estimated glomerular filtration rate; HTN, hypertension; CAD, coronary artery disease; CHF, congestive heart failure; ACEI, angiotensin converting enzyme inhibitor; ARB, angiotensin receptor blocker; LVEF, left ventricular ejection fraction; LVd, left ventricular diastolic diameter; LVMi, left ventricular mass index (corrected for body surface area); PAP, pulmonary artery pressure; RV, right ventricle

### Mortality

8780 patients died during the follow-up: 2118 (24% of deaths) among patients with an eGFR ≥90 (6.4 events per 100 patients-years), 3456 (39% of deaths) among patients with eGFR between 60 and 89 (7.2 events per 100 patients-years), and 3206 (37% of deaths) among patients with eGFR < 60 ml/min/1.73 m^2^ (13.6 events per 100 patients-years).

In adjusted models, LV ejection fraction group, LV diameter quartile, pulmonary artery pressure quartile, and RV parameters were independently associated with mortality (Table 3).

### Sensitivity analyses

Adjusted results were qualitatively similar in analyses of the 13,130 outpatients (Additional file 7: Table S7). Results were also similar in those without baseline CHF (*n* = 21,036, Additional file 8: Table S8), except that RV hypertrophy was not significantly associated with mortality, and in patients with available follow-up renal tests or codes (*n* = 20,008, Additional file 9: Table S9).

## Discussion

We assessed echocardiographic characteristics of patients with preserved renal function and mild or moderate CKD in a cohort of individuals referred for echocardiography. We found that patients with CKD have a higher prevalence of structural and functional LV and RV abnormalities, which are already apparent in early CKD (eGFR 60–89 ml/min/1.73m^2^). We also identified that RV systolic function or dilation and pulmonary artery pressure are independently associated with a composite renal outcome of serum creatinine doubling, dialysis, or transplantation. Both RV and LV echocardiographic features were associated with mortality.

Park et al. analyzed the CRIC population and found that decreasing renal function was associated with LV hypertrophy, abnormal LV geometry, diastolic dysfunction, but not with systolic dysfunction [5]. Gori et al. described cardiac structure and function in 217 individuals with heart failure with preserved ejection fraction from the PARAMOUNT trial [3]. They found that renal impairment was associated with abnormal LV geometry, higher LV mass or LV mass index, and lower mid-wall fractional shortening [3]. Creatinine clearance was also identified as an independent predictor of heart failure with preserved ejection fraction in a retrospective study of patients with hypertension and preserved ejection fraction [6]. In a cohort of post-myocardial infarction patients from the VALIANT trial, worse renal function was associated with smaller LV, larger left atrial volume, and higher LV mass index [7]. In contrast, in hypertensive patients with asymptomatic diastolic dysfunction from the VALIDD and EXCEED trials, eGFR group was not associated with echocardiographic measures of LV structure and function [8].

Our findings confirm most of these observations in a much larger population including patients with and without CHF. We also report a significant association of worse renal function with LV systolic impairment, and we expand the previous findings by looking at RV parameters and their association with CKD.

We found that only pulmonary artery pressure and parameters related to the RV are associated with CKD progression. The AASK study addressed this issue in African-American patients with pre-existing non-diabetic hypertensive CKD and showed that LV mass index was not associated with a combined outcome of doubling of creatinine or end-stage renal disease [9]. On the contrary, a retrospective analysis of the Jackson Heart Study, also in an African-American population, showed a significant association of LV mass, but not LV ejection fraction or pulmonary artery pressure, with a composite outcome of eGFR decline > 30% or progression to end-stage renal disease [10]. A small cohort study in stage 3–5 CKD patients demonstrated that concentric LV hypertrophy was associated with progression to dialysis, whereas LV ejection fraction and increased left atrium diameter were associated with CKD progression, defined as eGFR decline > 3 ml/min/1.73m^2^ annually [11, 12]. In addition, a study in 300 elderly patients showed that LV hypertrophy was independently associated with eGFR decline > 5 ml/min/1.73m^2^ per year [13]. In the ESCAPE trial, a significant correlation was identified between baseline creatinine levels and right atrial pressure levels in patients admitted with heart failure [14]. Park et al., using magnetic resonance imaging, demonstrated that LV mass and concentricity, a measurement of abnormal cardiac size, were associated with eGFR decline and incident CKD [15].

Discrepancies with our findings may reflect differences in baseline renal and heart function, demographic characteristics and the use of a clinical cohort in our study compared to highly selected prospective research or trial cohorts in the prior studies. Furthermore, renal outcomes were not defined in the same way. Lack of association between LV mass index and renal outcomes or mortality in our study may be due to values below hypertrophic levels for the majority of individuals. LV mass could be more reliably assessed with more advanced techniques, such as magnetic resonance imaging. Similarly, lack of association between pulmonary artery pressure and renal outcomes in the Jackson Heart Study may be due to relatively normal pulmonary artery pressure values among study participants.

Our findings extend these findings by demonstrating a significant, independent association between RV diameter or function and CKD progression in a population that primarily did not have CHF. Worsening RV function and elevated pulmonary artery pressures may be associated with higher renal vein pressures, a purported mechanism underlying the cardiorenal syndrome [16, 17]. Similarly, salt and water retention in the setting of decreasing eGFR may contribute to volume overload and increased renal vein pressures that may impair renal perfusion and cause ischemia-mediated sclerotic changes. On the contrary, LV dysfunction may not significantly impact renal function provided that renal perfusion pressure remains in an acceptable range. However, these findings are only hypothesis generating and need to be prospectively confirmed. Finally, we also report a significant association between mortality and abnormal RV structure or function. No prior data exist on the association between right heart parameters and mortality in patients with CKD, to our knowledge.

Our study has several limitations. It is a retrospective study of patients referred for echocardiography and may not reflect the complete spectrum of patients with preserved renal function or mild eGFR impairment since sicker patients are more likely to be referred for echocardiography and those with more advanced cardiopulmonary disease are more likely to have RV dysfunction. Furthermore, no information was available on the indication for the echocardiogram. Echocardiograms were ordered to answer specific clinical questions rather than to provide systemic assessment of cardiac structure and they were interpreted by multiple clinicians rather than by a core lab, raising the possibility of confounding introduced by wide inter-reader variability between sonographers. In particular, RV assessment is fraught with potential errors of judgment and high degree of inter-observer variability. Additionally, tissue Doppler imaging results, diastolic function, and valve structure and function were not extractable for most patients.

Index echocardiograms might have been conducted in the setting of decompensated CHF and may not reflect baseline cardiac status or could have been followed by acute changes in kidney function. However, our findings were qualitatively similar in a subgroup analysis including only outpatient echocardiograms in which these issues would be less likely. We similarly cannot rule out the possibility that addition of renin-angiotensin-system agents in individuals with more severe echocardiographic changes partly accounted for associations with CKD progression, although our results were adjusted for use of these agents at baseline. Furthermore, the clinical indication of the echocardiogram may have already caused changes in eGFR. Capture of medication changes during follow-up is not readily available to us at this time. Finally, there were no BP or proteinuria values available for most patients in this dataset, and no information was available on the cause of death. Nevertheless, a sample size several times larger than all prior cohorts, assessment of multiple echocardiographic parameters, and most importantly a sufficient number of outcomes to be powered to examine associations with CKD progression and mortality represent unique strengths of our analysis.

More studies are needed to prospectively confirm the identified associations and carefully evaluate the right heart with comprehensive assessment of the RV including lateral tricuspid annular systolic velocity and acceleration, fractional area and long-axis change, tricuspid annular systolic excursion, myocardial performance index, and lateral free wall peak systolic strain, as well as inferior vena cava diameter and distensibility [18, 19]. These results also merit analysis in the setting of advanced CKD (eGFR < 30 ml/min/1.73m^2^).

## Conclusions

In conclusion, structural and functional abnormalities of the right and left ventricle are more common in individuals with mild or moderate renal impairment and associated with mortality while RV function and structure are independently associated with CKD progression even in early stage CKD. Prospective studies with quantitative assessment of the RV are needed to validate our observations and better understand the underlying mechanisms.

## Additional files

Additional file 1: **Table S1.** Condition specific diagnostic codes (International Classification of Diseases, 9th revision) (DOCX 92 kb).

Additional file 2: **Table S2.** Crude and adjusted assessment of echocardiographic parameters and estimated glomerular filtration rate (DOCX 17 kb).

Additional file 3: **Table S3.** Baseline characteristics of patients with CHF at baseline (DOCX 18 kb).

Additional file 4: **Table S4.** Echocardiographic characteristics of patients with CHF at baseline (DOCX 17 kb).

Additional file 5: **Table S5.** Baseline characteristics of patients without CHF at baseline (DOCX 17 kb).

Additional file 6: **Table S6.** Echocardiographic characteristics of patients without CHF at baseline (DOCX 16 kb).

Additional file 7: **Table S7.** Adjusted associations of echocardiographic parameters with composite renal outcomes and mortality in outpatients (DOCX 16 kb).

Additional file 8: **Table S8.** Adjusted associations of echocardiographic parameters with composite renal outcomes and mortality in patients without known CHF at baseline (DOCX 16 kb)

Additional file 9: **Table S9.** Adjusted associations of echocardiographic parameters with composite renal outcomes and mortality in patients with available follow-up creatinine values or diagnostic codes (DOCX 16 kb).

## Abbreviations

ACEI: angiotensin converting enzyme inhibitors
ARB: angiotensin receptor blockers
BNP: brain natriuretic peptide
CHF: congestive heart failure
CKD: chronic kidney disease
eGFR: estimated glomerular filtration rate
FDA: Food and Drug Administration
LV: left ventricular
RPDR: Research Patient Data registry
RV: right ventricular
