# Supplementary material for: Echocardiographic parameters and renal outcomes in patients with preserved renal function, and mild- moderate CKD
Source: BMC Nephrol. 2018 Jul 11;19:176. doi: 10.1186/s12882-018-0975-5 (PMC6042465; doi:10.1186/s12882-018-0975-5)
Supplement: Supplementary file 1 — Table S1. Condition specific diagnostic codes (International Classification of Diseases, 9th revision) (DOCX 92 kb). [file 12882_2018_975_MOESM1_ESM.docx]

**Supplemental material**

**Echocardiographic parameters and renal outcomes in patients with preserved renal function, and mild- moderate CKD**

Thomas A. Mavrakanas, Aisha Khattak, Karandeep Singh, David M. Charytan

**Supplemental Table 1** Condition specific diagnostic codes (International Classification of Diseases, 9^th^ revision)

**Supplemental Table 2** Crude and adjusted assessment of echocardiographic parameters and estimated glomerular filtration rate

**Supplemental** **Table 3** Baseline characteristics of the patients with CHF at baseline

**Supplemental** **Table 4** Echocardiographic characteristics of patients with CHF at baseline

**Supplemental** **Table 5** Baseline characteristics of the patients without CHF at baseline

**Supplemental** **Table 6** Echocardiographic characteristics of patients without CHF at baseline

**Supplemental Table 7** Adjusted associations of echocardiographic parameters with composite renal outcomes and mortality in outpatients

**Supplemental Table 8** Adjusted associations of echocardiographic parameters with composite renal outcomes and mortality in patients without known CHF at baseline

**Supplemental Table 9** Adjusted associations of echocardiographic parameters with composite renal outcomes and mortality in patients with available follow-up creatinine values or diagnostic codes

**Supplemental Table 1** Condition specific diagnostic codes (International Classification of Diseases, 9^th^ revision)

| **Condition** | **Diagnostic code** |
| --- | --- |
| Hypertension | 401.x, 796.2 |
| Diabetes mellitus | 249.xx, 250.xx |
| Coronary artery disease | 410.xx, 412, 414.x(x) |
| Congestive heart failure | 402.01, 402.11, 402.91, 404.01, 404.03, 404.11, 404.13, 404.91, 404.93, 428. x(x) |
| Chronic obstructive pulmonary disease | 491.x, 492.x |
| Pulmonary embolism (acute or chronic) | 415.11, 415.13, 415.19, 416.2 |
| Dialysis | 585.6 |
| Kidney transplantation | V42.0, 996.81 |
