# Supplementary material for: Echocardiographic parameters and renal outcomes in patients with preserved renal function, and mild- moderate CKD
Source: BMC Nephrol. 2018 Jul 11;19:176. doi: 10.1186/s12882-018-0975-5 (PMC6042465; doi:10.1186/s12882-018-0975-5)
Supplement: Supplementary file 2 — Table S2. Crude and adjusted assessment of echocardiographic parameters and estimated glomerular filtration rate (DOCX 17 kb). [file 12882_2018_975_MOESM2_ESM.docx]

**Supplemental Table 2** Crude and adjusted assessment estimated glomerular filtration rate with individual echocardiographic endpoints

| **Outcome** | **Univariate** | | |  | **Multivariate** | | |  |
| --- | --- | --- | --- | --- | --- | --- | --- | --- |
|  | β | **95% CI** | **p** | **R^2^** | β | **95% CI** | **p** | **R^2^** |
| ln LVM | -0.026 | -0.029, -0.023 | <0.001 | 0.028 | -0.015 | -0.018, -0.012 | <0.001 | 0.247 |
| ln LVMi | -0.030 | -0.033, -0.027 | <0.001 | 0.047 | -0.010 | -0.014, -0.006 | <0.001 | 0.184 |
| LAd | -0.083 | -0.087, -0.078 | <0.001 | 0.061 | -0.041 | -0.047, -0.036 | <0.001 | 0.179 |
| ln PAP | -0.040 | -0.043, -0.038 | <0.001 | 0.056 | -0.010 | -0.013, -0.006 | <0.001 | 0.128 |
| **Binary outcome** | **OR** | **95% CI** | **p** |  | **OR** | **95% CI** | **p** |  |
| Reduced LVEF | 0.85 | 0.84, 0.86 | <0.001 | 0.038 | 0.93 | 0.91, 0.94 | <0.001 | 0.261 |
| Elevated RAP | 0.87 | 0.85, 0.90 | <0.001 | 0.020 | 0.92 | 0.89, 0.96 | <0.001 | 0.087 |
| RWMA | 0.81 | 0.79, 0.83 | <0.001 | 0.041 | 0.97 | 0.94, 1.00 | 0.05 | 0.256 |
| Impaired RV systolic function | 0.85 | 0.83, 0.86 | <0.001 | 0.026 | 0.86 | 0.84, 0.88 | <0.001 | 0.172 |
| RV hypertrophy | 0.84 | 0.81, 0.87 | <0.001 | 0.021 | 0.87 | 0.82, 0.91 | <0.001 | 0.054 |
| RV dilation | 0.89 | 0.88, 0.91 | <0.001 | 0.014 | 0.91 | 0.89, 0.93 | <0.001 | 0.068 |

The association between the echocardiographic parameters and eGFR (per 10 ml/min.1.73 m^2^) was studied using linear or binary logistic regression. The following parameters were entered in the multivariate model: age, sex, race, history of hypertension, diabetes, coronary disease, and heart failure.

β, beta coefficient; CI, confidence interval; ln, natural logarithm; LVM, left ventricular mass (area-length method); LVMi, left ventricular mass index (corrected for body surface area); LAd, left atrium diameter; PAP, pulmonary arterial pressure; OR, odds ratio; LVEF, left ventricular ejection fraction; RAP, right atrial pressure; RWMA, regional wall motion abnormalities; RV, right ventricle.
