# Supplementary material for: Echocardiographic parameters and renal outcomes in patients with preserved renal function, and mild- moderate CKD
Source: BMC Nephrol. 2018 Jul 11;19:176. doi: 10.1186/s12882-018-0975-5 (PMC6042465; doi:10.1186/s12882-018-0975-5)
Supplement: Supplementary file 3 — Table S3. Baseline characteristics of patients with CHF at baseline (DOCX 18 kb). [file 12882_2018_975_MOESM3_ESM.docx]

**Supplemental Table 3** Baseline characteristics of the patients with CHF at baseline

| Characteristic | eGFR 90-120 | | eGFR 60-89 | | eGFR 30-59 | |
| --- | --- | --- | --- | --- | --- | --- |
|  | N | Result | N | Result | N | Result |
| Outpatients | 1733 | 438 (25%) | 3369 | 868 (26%) | 3081 | 720 (23%) |
| Age (years) | 1733 | 53 ± 13.6 | 3369 | 67 ± 13.6 | 3081 | 73 ± 12.2 |
| Male sex | 1733 | 961 (56%) | 3369 | 1963 (58%) | 3081 | 1635 (53%) |
| African-American | 1733 | 231 (13%) | 3369 | 279 (8%) | 3081 | 236 (8%) |
| Hypertension | 1733 | 765 (44%) | 3369 | 2024 (60%) | 3081 | 1882 (61%) |
| Diabetes | 1733 | 361 (21%) | 3369 | 878 (26%) | 3081 | 1097 (36%) |
| CAD | 1733 | 789 (46%) | 3369 | 2051 (61%) | 3081 | 2094 (68%) |
| COPD | 1733 | 87 (5%) | 3369 | 182 (5%) | 3081 | 210 (7%) |
| PE | 1733 | 115 (7%) | 3369 | 142 (4%) | 3081 | 121 (4%) |
| ACEI | 1647 | 466 (28%) | 3192 | 1239 (39%) | 2937 | 1227 (42%) |
| ARB | 1647 | 94 (6%) | 3192 | 334 (11%) | 2937 | 418 (14%) |
| β-blocker | 1647 | 985 (60%) | 3192 | 2253 (71%) | 2937 | 2135 (73%) |
| Statin | 1647 | 613 (37%) | 3192 | 1713 (54%) | 2937 | 1818 (62%) |
| Aspirin | 1647 | 797 (48%) | 3192 | 1959 (61%) | 2937 | 1902 (65%) |
| Clopidogrel | 1647 | 187 (11%) | 3192 | 485 (15%) | 2937 | 488 (17%) |
| Warfarin | 1647 | 261 (16%) | 3192 | 767 (24%) | 2937 | 745 (25%) |
| Creatinine (μmol/l) | 1733 | 65  (55-74) | 3369 | 84  (72-95) | 3081 | 119  (104-140) |
| eGFR (ml/min.1.73 m^2^) | 1733 | 103 ± 11 | 3369 | 75 ± 9 | 3081 | 46 ± 9 |
| UACR (mg/mmol) | 63 | 3 (1-16) | 151 | 3 (1-12) | 198 | 7 (2-33) |
| Hb (g/l) | 1364 | 120 ± 24 | 2452 | 122 ± 22 | 2132 | 116 ± 20 |
| Albumin (g/l) | 1402 | 36 ± 7 | 2750 | 37 ± 6 | 2532 | 36 ± 6 |
| K^+^ (mmol/l) | 1712 | 4.0 ± 0.5 | 3315 | 4.0 ± 0.5 | 3039 | 4.1 ± 0.5 |
| Ca^++^ (mmol/l) | 1528 | 2.18 ± 0.18 | 3007 | 2.20 ± 0.18 | 2747 | 2.20 ± 0.18 |
| Phos (mmol/l) | 950 | 1.0 (0.8- 1.2) | 1605 | 1.0 (0.9- 1.2) | 1623 | 1.1 (0.9- 1.3) |
| BNP (ng/l) | 404 | 169 (49- 440) | 979 | 321 (130- 697) | 1145 | 438 (196- 911) |

Results are presented as number (percentage), mean ± standard deviation, or median (interquartile range). eGFR, estimated glomerular filtration rate; N, number of patients with available data; CAD, coronary artery disease; CHF, congestive heart failure; COPD, chronic obstructive lung disease; PE, pulmonary embolism (acute or chronic); ACEI, angiotensin converting enzyme inhibitor; ARB, angiotensin receptor blocker; UACR, urine albumin to creatinine ratio; Hb, hemoglobin; K^+^, potassium; Ca^++^, calcium; P, phosphorus; BNP, brain natriuretic peptide. Percentages are within eGFR group and exclude missing values. The three eGFR groups were statistically different (P value for trend <0.05 for all parameters).
