# Supplementary material for: Echocardiographic parameters and renal outcomes in patients with preserved renal function, and mild- moderate CKD
Source: BMC Nephrol. 2018 Jul 11;19:176. doi: 10.1186/s12882-018-0975-5 (PMC6042465; doi:10.1186/s12882-018-0975-5)
Supplement: Supplementary file 4 — Table S4. Echocardiographic characteristics of patients with CHF at baseline (DOCX 17 kb). [file 12882_2018_975_MOESM4_ESM.docx]

**Supplemental Table 4** Echocardiographic characteristics of patients with CHF at baseline

|  | eGFR 90-120 | | eGFR 60-89 | | eGFR 30-59 | |
| --- | --- | --- | --- | --- | --- | --- |
| Characteristic | N | Result | N | Result | N | Result |
| Preserved EF | 1602 | 918 (57%) | 3132 | 1592 (51%) | 2880 | 1336 (46%) |
| LVEF (%) | 1602 | 55 (40-60) | 3132 | 55 (35-60) | 2880 | 50 (30-60) |
| LVd (cm) | 1422 | 4.92 ± 0.97 | 2817 | 4.95 ± 0.99 | 2622 | 4.97 ± 1.06 |
| LVPW (cm) | 1054 | 1.03 ± 0.23 | 1945 | 1.08 ± 0.24 | 1742 | 1.11 ± 0.27 |
| IVSd (cm) | 1306 | 1.07 ± 0.25 | 2576 | 1.13 ± 0.27 | 2425 | 1.16 ± 0.27 |
| LVM (g) | 869 | 189 (141-238) | 1417 | 198 (151-253) | 1250 | 204 (158-264) |
| LVMi (g/m^2^) | 600 | 97 (77-122) | 1027 | 103 (83-133) | 874 | 113 (90-142) |
| LAd (cm) | 1346 | 3.92 ± 0.86 | 2705 | 4.23 ± 0.84 | 2542 | 4.38 ± 0.84 |
| PAP (mmHg) | 973 | 25 (20-34) | 2257 | 28 (22-37) | 2203 | 31 (23-41) |
| RAP >6 cm H_2_O | 688 | 91 (13%) | 1330 | 260 (20%) | 1146 | 288 (25%) |
| Impaired RV systolic function | 1419 | 221 (16%) | 2801 | 518 (19%) | 2563 | 585 (23%) |
| Increased RV diameter | 1471 | 251 (17%) | 2938 | 522 (18%) | 2675 | 636 (24%) |
| RV hypertrophy | 753 | 33 (4%) | 1493 | 69 (5%) | 1278 | 118 (9%) |
| ARd (cm) | 1262 | 3.14 ± 0.48 | 2575 | 3.21 ± 0.49 | 2425 | 3.18 ± 0.50 |
| RWMA | 837 | 137 (16%) | 1511 | 351 (23%) | 1282 | 346 (27%) |

Results are presented as number (percentage), mean ± standard deviation, or median (interquartile range). N, number of patients with available data; LVEF, left ventricular ejection fraction; EF, ejection fraction; LVd, left ventricular diastolic diameter; LVPW, left ventricular posterior wall thickness; IVSd, intraventricular septal thickness in diastole; LVM, left ventricular mass (area-length method); LVMi, left ventricular mass index (corrected for body surface area); LAd, left atrium diameter; PAP, pulmonary arterial pressure; RAP, right atrial pressure; RV, right ventricle; ARd, aortic root diameter; RWMA, regional wall motion abnormalities. The three eGFR groups were statistically different (P value for trend <0.05 for all parameters except LVd and ARd).
