# Supplementary material for: Echocardiographic parameters and renal outcomes in patients with preserved renal function, and mild- moderate CKD
Source: BMC Nephrol. 2018 Jul 11;19:176. doi: 10.1186/s12882-018-0975-5 (PMC6042465; doi:10.1186/s12882-018-0975-5)
Supplement: Supplementary file 5 — Table S5. Baseline characteristics of patients without CHF at baseline (DOCX 17 kb). [file 12882_2018_975_MOESM5_ESM.docx]

**Supplemental Table 5** Baseline characteristics of the patients without CHF at baseline

| Characteristic | eGFR 90-120 | | eGFR 60-89 | | eGFR 30-59 | |
| --- | --- | --- | --- | --- | --- | --- |
|  | N | Result | N | Result | N | Result |
| Outpatients | 8131 | 4120 (51%) | 9362 | 5256 (56%) | 3543 | 1728 (49%) |
| Age (years) | 8131 | 49 ± 14 | 9362 | 63 ± 13 | 3543 | 72 ± 12 |
| Male sex | 8131 | 3836 (47%) | 9362 | 4645 (50%) | 3543 | 1646 (47%) |
| African-American | 8131 | 937 (12%) | 9362 | 670 (7%) | 3543 | 287 (8%) |
| Hypertension | 8131 | 2715 (33%) | 9362 | 4860 (52%) | 3543 | 2209 (62%) |
| Diabetes | 8131 | 947 (12%) | 9362 | 1429 (15%) | 3543 | 805 (23%) |
| CAD | 8131 | 2298 (28%) | 9362 | 3933 (42%) | 3543 | 1668 (47%) |
| COPD | 8131 | 199 (2%) | 9362 | 272 (3%) | 3543 | 109 (3%) |
| PE | 8131 | 405 (5%) | 9362 | 310 (3%) | 3543 | 136 (4%) |
| ACEI | 7306 | 1148 (16%) | 8450 | 2039 (24%) | 3238 | 995 (31%) |
| ARB | 7306 | 236 (3%) | 8450 | 594 (7%) | 3238 | 392 (12%) |
| β-blocker | 7306 | 2633 (36%) | 8450 | 3937 (47%) | 3238 | 1767 (55%) |
| Statin | 7306 | 1685 (23%) | 8450 | 3364 (40%) | 3238 | 1499 (46%) |
| Aspirin | 7306 | 2196 (30%) | 8450 | 3328 (39%) | 3238 | 1471 (45%) |
| Clopidogrel | 7306 | 472 (7%) | 8450 | 840 (10%) | 3238 | 356 (11%) |
| Warfarin | 7306 | 679 (9%) | 8450 | 1085 (13%) | 3238 | 423 (13%) |
| Creatinine (mmol/l) | 8131 | 65  (57-74) | 9362 | 82  (72-93) | 3543 | 112  (97-130) |
| eGFR (ml/min/1.73m^2^) | 8131 | 105 ± 12 | 9362 | 76 ± 8 | 3543 | 49 ± 8 |
| UACR (mg/ mmol) | 285 | 2 (1-6) | 393 | 2 (1-6) | 274 | 4 (1-20) |
| Hb (g/l) | 5751 | 124 ± 22 | 6006 | 130 ± 20 | 2169 | 120 ± 21 |
| Albumin (g/l) | 5929 | 39 ± 7 | 6511 | 40 ± 6 | 2651 | 38 ± 7 |
| K^+^ (mmol/l) | 7810 | 3.9 ± 0.4 | 8997 | 4.0 ± 0.4 | 3409 | 4.1 ± 0.5 |
| Ca^++^ (mmol/l) | 6963 | 2.23 ± 0.18 | 7954 | 2.25 ± 0.15 | 3049 | 2.23 ± 0.18 |
| Phos (mmol/l) | 3024 | 1.0 (0.8-1.2) | 2583 | 1.0 (0.9-1.2) | 1294 | 1.1 (0.9-1.2) |
| BNP (ng/l) | 502 | 53 (20-140) | 631 | 74 (30-187) | 374 | 143 (64-359) |

Results are presented as number (percentage), mean ± standard deviation, or median (interquartile range). eGFR, estimated glomerular filtration rate; N, number of patients with available data; CAD, coronary artery disease; CHF, congestive heart failure; COPD, chronic obstructive lung disease; PE, pulmonary embolism (acute or chronic); ACEI, angiotensin converting enzyme inhibitor; ARB, angiotensin receptor blocker; UACR, urine albumin to creatinine ratio; Hb, hemoglobin; K^+^, potassium; Ca^++^, calcium; P, phosphorus; BNP, brain natriuretic peptide. Percentages are within eGFR group and exclude missing values. The three eGFR groups were statistically different (P value for trend <0.05 for all parameters except sex, hemoglobin, and albumin).
