# Supplementary material for: Echocardiographic parameters and renal outcomes in patients with preserved renal function, and mild- moderate CKD
Source: BMC Nephrol. 2018 Jul 11;19:176. doi: 10.1186/s12882-018-0975-5 (PMC6042465; doi:10.1186/s12882-018-0975-5)
Supplement: Supplementary file 6 — Table S6. Echocardiographic characteristics of patients without CHF at baseline (DOCX 16 kb). [file 12882_2018_975_MOESM6_ESM.docx]

**Supplemental Table 6** Echocardiographic characteristics of patients without CHF at baseline

|  | eGFR 90-120 | | eGFR 60-89 | | eGFR 30-59 | |
| --- | --- | --- | --- | --- | --- | --- |
| Characteristic | N | Result | N | Result | N | Result |
| Preserved EF | 7559 | 6686 (88%) | 8598 | 7383 (86%) | 3312 | 2722 (82%) |
| LVEF (%) | 7559 | 60 (55-60) | 8598 | 60 (55-60) | 3312 | 60 (55-60) |
| LVd (cm) | 6154 | 4.60 ± 0.64 | 6745 | 4.52 ± 0.67 | 2789 | 4.45 ± 0.71 |
| LVPW (cm) | 4193 | 0.97 ± 0.21 | 4196 | 1.02 ± 0.22 | 1689 | 1.07 ± 0.23 |
| IVSd (cm) | 5612 | 1.01 ± 0.23 | 6183 | 1.08 ± 0.24 | 2550 | 1.14 ± 0.25 |
| LVM (g) | 2984 | 155  (123-195) | 2812 | 165  (129-207) | 1127 | 170  (135-217) |
| LVMi (g/m^2^) | 2257 | 82 (68-99) | 2177 | 87 (71-107) | 831 | 93 (75-114) |
| LAd (cm) | 5953 | 3.60 ± 0.66 | 6572 | 3.79 ± 0.72 | 2703 | 3.92 ± 0.77 |
| PAP (mmHg) | 4336 | 21 (17-27) | 5128 | 23 (19-29) | 2156 | 26 (21-33) |
| RAP >6 cm H_2_O | 3143 | 192 (6%) | 3070 | 189 (6%) | 1171 | 84 (7%) |
| Impaired RV systolic function | 6391 | 209 (3%) | 7086 | 271 (4%) | 2813 | 132 (5%) |
| Increased RV diameter | 6511 | 477 (7%) | 7210 | 592 (8%) | 2880 | 271 (9%) |
| RV hypertrophy | 4237 | 84 (2%) | 4395 | 102 (2%) | 1562 | 54 (4%) |
| ARd (cm) | 5684 | 3.08 ± 0.47 | 6352 | 3.17 ± 0.47 | 2627 | 3.18 ± 0.46 |
| RWMA | 5483 | 211 (4%) | 5855 | 367 (6%) | 2095 | 194 (9%) |

Results are presented as number (percentage), mean ± standard deviation, or median (interquartile range). N, number of patients with available data; LVEF, left ventricular ejection fraction; EF, ejection fraction; LVd, left ventricular diastolic diameter; LVPW, left ventricular posterior wall thickness; IVSd, intraventricular septal thickness in diastole; LVM, left ventricular mass (area-length method); LVMi, left ventricular mass index (corrected for body surface area); LAd, left atrium diameter; PAP, pulmonary arterial pressure; RAP, right atrial pressure; RV, right ventricle; ARd, aortic root diameter; RWMA, regional wall motion abnormalities. Percentages exclude missing values. The three eGFR groups were statistically different (P value for trend <0.01 for all parameters except RAP).
