# Supplementary material for: Echocardiographic parameters and renal outcomes in patients with preserved renal function, and mild- moderate CKD
Source: BMC Nephrol. 2018 Jul 11;19:176. doi: 10.1186/s12882-018-0975-5 (PMC6042465; doi:10.1186/s12882-018-0975-5)
Supplement: Supplementary file 7 — Table S7. Adjusted associations of echocardiographic parameters with composite renal outcomes and mortality in outpatients (DOCX 16 kb). [file 12882_2018_975_MOESM7_ESM.docx]

**Supplemental Table 7** Adjusted associations of echocardiographic parameters with composite renal outcomes and mortality in outpatients (n=13,130)

|  | **Parameter** | **Adjusted HR (95% CI) - Renal outcomes** | **p** | **Adjusted HR (95% CI) - Mortality** | **p** |
| --- | --- | --- | --- | --- | --- |
| LVEF | Group 1 vs. 4 | 1.66 (0.88-3.12) | 0.12 | 1.46 (1.10-1.95) | 0.01 |
|  | Group 2 vs. 4 | 1.17 (0.68-2.00) | 0.57 | 1.42 (1.16-1.74) | 0.001 |
|  | Group 3 vs. 4 | 1.15 (0.80- 1.64) | 0.45 | 1.05 (0.91-1.21) | 0.50 |
| LVd | Quartile 2 vs. 1 | 0.58 (0.40-0.83) | 0.003 | 0.83 (0.73-0.95) | 0.006 |
|  | Quartile 3 vs. 1 | 0.86 (0.61-1.22) | 0.40 | 0.68 (0.59-0.79) | <0.001 |
|  | Quartile 4 vs. 1 | 0.88 (0.62-1.26) | 0.49 | 0.75 (0.65-0.87) | <0.001 |
| LVMi | Quartile 2 vs. 1 | 1.05 (0.50-2.19) | 0.89 | 0.81 (0.64-1.03) | 0.09 |
|  | Quartile 3 vs. 1 | 1.43 (0.70-2.92) | 0.32 | 0.98 (0.78-1.24) | 0.87 |
|  | Quartile 4 vs. 1 | 1.82 (0.88-3.80) | 0.11 | 0.88 (0.68-1.13) | 0.32 |
| PAP | Quartile 2 vs. 1 | 1.08 (0.72-1.64) | 0.71 | 1.08 (0.93-1.27) | 0.31 |
|  | Quartile 3 vs. 1 | 1.74 (1.14-2.66) | 0.01 | 1.21 (1.03-1.43) | 0.02 |
|  | Quartile 4 vs. 1 | 2.32 (1.51-3.58) | <0.001 | 1.66 (1.40-1.96) | <0.001 |
| RV systolic function | Reduced vs. preserved | 2.61 (1.67-4.08) | <0.001 | 1.77 (1.44-2.17) | <0.001 |
| RV hypertrophy | Present vs. absent | 1.01 (0.42-2.39) | 0.99 | 1.20 (0.87-1.65) | 0.27 |
| RV dilation | Present vs. absent | 1.84 (1.27-2.65) | 0.001 | 1.38 (1.17-1.62) | <0.001 |

Adjusted Cox models. Hazard ratios are adjusted for age, sex, race, baseline eGFR, history of hypertension, diabetes, CAD, or CHF, and use of ACEI and/or ARB. Composite renal outcome includes doubling of serum creatinine or initiation of maintenance dialysis or kidney transplantation. For LVEF, the following cutoffs were used: <25%, 25-39%, 40-54%, ≥55%. HR, hazards ratio; CI, confidence interval; AA, African American race; eGFR, estimated glomerular filtration rate; HTN, hypertension; CAD, coronary artery disease; CHF, congestive heart failure; ACEI, angiotensin converting enzyme inhibitor; ARB, angiotensin receptor blocker; LVEF, left ventricular ejection fraction; LVd, left ventricular diastolic diameter; LVMi, left ventricular mass index (corrected for body surface area); PAP, pulmonary arterial pressure; RV, right ventricle.
