# Supplementary material for: Echocardiographic parameters and renal outcomes in patients with preserved renal function, and mild- moderate CKD
Source: BMC Nephrol. 2018 Jul 11;19:176. doi: 10.1186/s12882-018-0975-5 (PMC6042465; doi:10.1186/s12882-018-0975-5)
Supplement: Supplementary file 8 — Table S8. Adjusted associations of echocardiographic parameters with composite renal outcomes and mortality in patients without known CHF at baseline (DOCX 16 kb) [file 12882_2018_975_MOESM8_ESM.docx]

**Supplemental Table 8** Adjusted associations of echocardiographic parameters with composite renal outcomes and mortality in patients without known CHF at baseline (N=21,036)

|  | **Parameter** | **Adjusted HR (95% CI) - Renal outcomes** | **p** | **Adjusted HR (95% CI) - Mortality** | **p** |
| --- | --- | --- | --- | --- | --- |
| LVEF | Group 1 vs. 4 | 0.55 (0.08-3.94) | 0.55 | 1.77 (1.24-2.52) | 0.002 |
|  | Group 2 vs. 4 | 1.50 (0.89-2.53) | 0.13 | 1.69 (1.43-2.00) | <0.001 |
|  | Group 3 vs. 4 | 0.92 (0.70-1.22) | 0.58 | 1.15 (1.06-1.26) | 0.001 |
| LVd | Quartile 2 vs. 1 | 0.73 (0.56-0.94) | 0.02 | 0.80 (0.74-0.87) | <0.001 |
|  | Quartile 3 vs. 1 | 0.95 (0.73-1.22) | 0.66 | 0.77 (0.71-0.84) | <0.001 |
|  | Quartile 4 vs. 1 | 1.01 (0.76-1.34) | 0.96 | 0.76 (0.69-0.84) | <0.001 |
| LVMi | Quartile 2 vs. 1 | 0.71 (0.45-1.13) | 0.15 | 0.89 (0.76-1.04) | 0.14 |
|  | Quartile 3 vs. 1 | 1.00 (0.65-1.55) | 1.00 | 0.91 (0.77-1.07) | 0.26 |
|  | Quartile 4 vs. 1 | 0.82 (0.49-1.37) | 0.44 | 0.91 (0.76-1.09) | 0.31 |
| PAP | Quartile 2 vs. 1 | 1.35 (1.00-1.82) | 0.05 | 1.11 (1.01-1.23) | 0.04 |
|  | Quartile 3 vs. 1 | 1.83 (1.33-2.51) | <0.001 | 1.26 (1.13-1.40) | <0.001 |
|  | Quartile 4 vs. 1 | 2.20 (1.57-3.08) | <0.001 | 1.77 (1.60-1.97) | <0.001 |
| RV systolic function | Reduced vs. preserved | 1.82 (1.21-2.72) | 0.004 | 1.59 (1.38-1.83) | <0.001 |
| RV hypertrophy | Present vs. absent | 1.17 (0.60-2.29) | 0.65 | 0.97 (0.77-1.23) | 0.83 |
| RV dilation | Present vs. absent | 1.22 (0.88-1.71) | 0.24 | 1.34 (1.21-1.49) | <0.001 |

Adjusted Cox models. Hazard ratios are adjusted for age, sex, race, baseline eGFR, history of hypertension, diabetes, CAD, and use of ACEI and/or ARB. Composite renal outcome includes doubling of serum creatinine or initiation of maintenance dialysis or renal transplantation. For LVEF, the following cutoffs were used: <25%, 25-39%, 40-54%, ≥55%. HR, hazards ratio; CI, confidence interval; AA, African American race; eGFR, estimated glomerular filtration rate; HTN, hypertension; CAD, coronary artery disease; CHF, congestive heart failure; ACEI, angiotensin converting enzyme inhibitor; ARB, angiotensin receptor blocker; LVEF, left ventricular ejection fraction; LVd, left ventricular diastolic diameter; LVMi, left ventricular mass index (corrected for body surface area); PAP, pulmonary arterial pressure; RV, right ventricle.
