# Supplementary material for: Echocardiographic parameters and renal outcomes in patients with preserved renal function, and mild- moderate CKD
Source: BMC Nephrol. 2018 Jul 11;19:176. doi: 10.1186/s12882-018-0975-5 (PMC6042465; doi:10.1186/s12882-018-0975-5)
Supplement: Supplementary file 9 — Table S9. Adjusted associations of echocardiographic parameters with composite renal outcomes and mortality in patients with available follow-up creatinine values or diagnostic codes (DOCX 16 kb). [file 12882_2018_975_MOESM9_ESM.docx]

**Supplemental Table 9** Adjusted associations of echocardiographic parameters with composite renal outcomes in patients with available follow-up creatinine values or diagnostic codes (N=20,008)

|  | **Parameter** | **Adjusted HR (95% CI) - Renal outcomes** | **p** |
| --- | --- | --- | --- |
| LVEF | Group 1 vs. 4 | 1.19 (0.89-1.61) | 0.24 |
|  | Group 2 vs. 4 | 1.06 (0.83-1.37) | 0.63 |
|  | Group 3 vs. 4 | 1.05 (0.87-1.27) | 0.63 |
| LVd | Quartile 2 vs. 1 | 0.77 (0.63-0.95) | 0.02 |
|  | Quartile 3 vs. 1 | 0.95 (0.77-1.17) | 0.63 |
|  | Quartile 4 vs. 1 | 1.02 (0.83-1.26) | 0.85 |
| LVMi | Quartile 2 vs. 1 | 0.72 (0.47-1.09) | 0.12 |
|  | Quartile 3 vs. 1 | 1.28 (0.88-1.84) | 0.19 |
|  | Quartile 4 vs. 1 | 0.85 (0.57-1.29) | 0.45 |
| PAP | Quartile 2 vs. 1 | 1.27 (0.99-1.63) | 0.06 |
|  | Quartile 3 vs. 1 | 1.69 (1.31-2.18) | <0.001 |
|  | Quartile 4 vs. 1 | 2.27 (1.78-2.90) | <0.001 |
| RV systolic function | Reduced vs. preserved | 1.52 (1.21-1.90) | <0.001 |
| RV hypertrophy | Present vs. absent | 1.20 (0.78-1.85) | 0.42 |
| RV dilation | Present vs. absent | 1.62 (1.34-1.96) | <0.001 |

Adjusted Cox models. Hazard ratios are adjusted for age, sex, race, baseline eGFR, history of hypertension, diabetes, CAD, or CHF, and use of ACEI and/or ARB. Composite renal outcome includes doubling of serum creatinine or initiation of maintenance dialysis or kidney transplantation. For LVEF, the following cutoffs were used: <25%, 25-39%, 40-54%, ≥55%. HR, hazards ratio; CI, confidence interval; AA, African American race; eGFR, estimated glomerular filtration rate; HTN, hypertension; CAD, coronary artery disease; CHF, congestive heart failure; ACEI, angiotensin converting enzyme inhibitor; ARB, angiotensin receptor blocker; LVEF, left ventricular ejection fraction; LVd, left ventricular diastolic diameter; LVMi, left ventricular mass index (corrected for body surface area); PAP, pulmonary arterial pressure; RV, right ventricle.
